# Surgery corrects asynchrony of ribcage secondary to extra-thoracic tumor but leads to expiratory dysfunction during exercise

**DOI:** 10.1186/s13019-015-0355-1

**Published:** 2015-12-18

**Authors:** Ghazi Elshafie, Andrea Aliverti, Ludovica Pippa, Prem Kumar, Maninder Kalkat, Babu Naidu

**Affiliations:** 1 Department of Thoracic Surgery, Heart of England NHS foundation trust, Bordesley Green East, Birmingham, West Midlands B9 5SS UK; 2 Dipartimento di Elettronica, Informazione e Bioingegneria, Politecnico di Milano, P.zza L. da Vinci, 32, 20133 Milan, Italy; 3 School of Clinical and Experimental Medicine, The Medical School, Vincent Drive University of Birmingham, Birmingham, B15 2TT UK

**Keywords:** Chest wall mechanics, Chest wall cancer, Optoelectronic plethysmography

## Abstract

**Background:**

The effect of chest wall tumours on chest wall mechanics is uncertain even less is known about the effects of resection and reconstruction. Our aim is to study how chest wall mechanics are altered in chest wall sarcoma and to determine the effect of chest wall reconstruction on chest wall kinetics.

**Case presentation:**

Using Optoelectronic Plethysmography (OEP), total and regional chest wall volumes were measured in a patient with unilateral extra-thoracic chest wall sarcoma, before and 5 months after resection and reconstruction, during quiet breathing and exercise using cycle ergometry.

During quiet breathing the unilateral tumour was associated with reduced in motion of the lower rib cage and abdominal compartments on both sides of the chest as well as asynchronous motion of the contralateral lower rib cage. Surgery corrected these abnormalities in quiet breathing. But during exercise there was a reduction in the upper rib cage motion compared to pre-operative measures from 0.43+/−0.06 to 0.36 +/− 0.02 L postoperatively (*p* <0.05). This impairment was characterised by a significant increase in the end expiratory volume on the operated side of the chest 5 months after surgery by 6.5 +/− 0.6 and 5.7 +/− 0.7 % during 50 and 100 % exercise respectively (*p* <0.0001) a finding that was not replicated in the non-operated side.

**Conclusion:**

This physiological study demonstrates the negative effect of chest wall tumours on global chest wall mechanics during quiet breathing and exercise and shows that surgery reverses this abnormality, but only at rest.

## Background

Sarcomas are rare tumours where dyspnoea can be a presenting symptom [1] whether this a result of dysfunctional chest wall motion in these patients is yet to be known. Furthermore ideal reconstructive prosthesis is still a matter of debate and is largely depend on the surgeon’s preference. The effect of type of prosthesis on chest wall dynamics has not been reported before. In this case study we describe for the first time in detail how chest wall mechanics are altered in a patient with isolated extra-thoracic chest wall sarcoma who undergoes chest wall resection and reconstruction using non-rigid prosthesis both during quiet breathing and exercise.

## Case presentation

### Methods

**Fig. 1:**
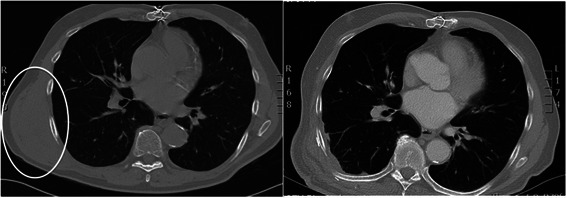
These two CT scans of the chest illustrate the preoperative chest wall sarcoma (circle - left) and 5 month postoperatively the prolene mesh reconstruction (right)

Using Optoelectronic Plethysmography (OEP), total and regional chest wall volumes were measured using eight infrared cameras, tracking the displacement of 89 chest wall markers on the patient’s chest, calculating compartmental motion and volumes [2]. Our subject is a 76 year old male patient diagnosed with spindle cell sarcoma of the right postero-lateral extrathoracic aspect of the chest wall: the tumour was 12 cm × 8 cm in size lying deep and inferior to the right scapula extending down to the 10th rib and involving the 4th rib (Fig. 1). Surgery was performed by mobilizing the latissmus dorsi muscle of the tumour, the 4th rib and the serratus anterior muscle attached to the tumour were excised en bloc. The resulting defect was covered with a tightly secured prolene mesh and a rotated latissmus dorsi flap. Spirometry was performed prior to every chest wall motion capture. Chest wall motion was measured in an erect position during quiet breathing and exercise using cycle ergometry preoperatively and postoperatively at 5 months as previously described [2].

Data is presented as mean (±SD) for normally distributed data. Paired student t tests were used to compare data before and after surgery. A statistical significance of 0.05 was used for all analyses.

### Results

**Fig. 2:**
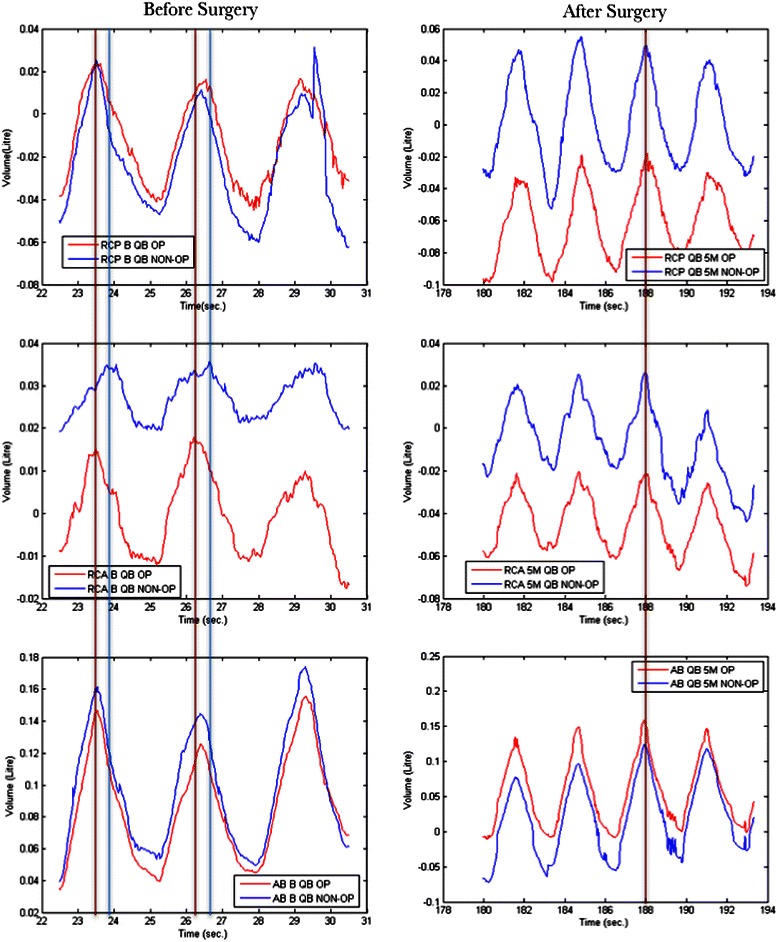
This graph demonstrates a breath-by-breath analysis examining the synchrony between chest compartments. The left panel shows pre-operative chest wall motion during quiet breathing, from top to bottom of the upper rib cage (RCP), lower rib cage (RCA) and the abdomen (AB) respectively. The red line represents the operated side and blue line the non-operated. The right panel shows motion in each compartment 5 months after surgery. The lower rib cage on the opposite side to the tumor side lagged by 56.8 +/− 15.1° out of phase compared to the side with the tumour. After surgery this asynchrony improved to 14.4 +/− 9.6° (*p* = 0.002)

During quiet breathing there was asynchronous lower rib cage compartment motion on the opposite side to the tumour compared to all other compartments. This asynchrony corrected when measured 5 months after surgery (Fig. 2).

Five months after surgery during quiet breathing the overall tidal volume significantly improved by 23 +/− 22 % compared to the preoperative value (*p* <0.002). This was due to an increased contribution of the lower rib cage and abdominal compartments by 107 +/− 42 % (*p* <0.001) and 17 +/− 20 % (*p* <0.01) respectively, equally on both sides of the chest.

During exercise there was a 41 +/− 15 % reduction of the upper rib cage motion on the operated side 5 months after surgery (*p* <0.001) this was not replicated on the non-operated side.

Sub-analysis of the end expiratory (EEV) and inspiratory volumes during exercise showed that, the reduction of the motion in the upper rib cage on the operated side was due to an increase in the EEV on that side (Table 2). There was also a 55.6 % significant reduction of the expiratory reserve volume on that side of upper rib cage during the same time frame (*p* <0.05).

**Table 1:** Respiratory parameters measured using OEP during quiet breathing before and 5 months after right chest wall resection

	Preoperatively	5 months post op	
	Value (L)	Value (L)	*P*
Overall Tidal volume	0.45 +/− 0.07	0.52 +/− 0.03	0.002
Tidal Volume of upper rib cage	0.15 +/ 0.03	0.14 +/− 0.02	0.31
Tidal volume of lower rib cage	0.04 +/− 0.01	0.08 +/− 0.01	0.0001
Tidal volume of abdomen	0.26 +/− 0.05	0.3 +/− 0.02	0.01
Tidal volume of right lower rib cage (operated side)	0.03 +/− 0.01	0.04 +/− 0.01	0.0001
Tidal volume of left lower rib cage (non-operated side)	0.02 +/− 0.01	0.04 +/− 0.01	0.0001
Tidal volume of right chest (operated side)	0.22 +/− 0.04	0.25 +/− 0.02	0.005
Tidal volume of left chest (non-operated side)	0.23 +/− 0.04	0.27 +/− 0.02	0.0008

**Table 2:** The table shows the change in EEV volume of the RCP on both sides of chest during 50 and 100 % exercise before and 5 months after surgery (Neg : negative change). Its clear EEV of RCP on the operated side to increased after surgery during 50 and 100 % exercise suggesting a defective expiratory mechanism on the that side

		Change at 5 months	*P* Value
△ EEV on RCP operated side	50 % Exercise	6.5 +/− 0.6 %	0.0001
	100 % Exercise	5.7 +/− 0.7 %	0.0001
△ EEV on RCP non-operated side	50 % Exercise	Neg 1.6 +/− 1.1 %	0.0006
	100 % Exercise	Neg 1.2 +/− 0.8 %	0.0001

The forced expiratory volume in one second (FEV1) was 2.7 +/− 0.2 L litres preoperatively (99 % predicted) and the forced vital capacity (FVC) was 3.67 +/− 0.2 (108 % predicted). Five month after surgery there was a significant reduction in FEV1 to 2.38 +/− 0.2 L (*p* <0.05) but the FVC remained unchanged 3.69 +/− 0.3 L (p 0.06).

### Discussion

Our case report shows that an extra-thoracic chest wall sarcoma exerts a restrictive mass effect on regional chest wall motion resulting in asynchrony and reduced volumes in the lower rib and abdomen compartments due to the position of the tumour. Similar findings are described in individuals with restrictive pulmonary disease [3]. This defect improves following resection and reconstruction with a semi rigid taught prolene mesh. As the chest wall works as a unit the impairment is apparent on both sides of the chest though the tumour is unilateral. We are uncertain as to why it is the contralateral lower rib cage side that lags behind all other compartments but it may be due to a mechanical displacement of that part of the chest due to the presence of such a large tumour.

Removing the extra thoracic chest wall tumour and covering the defect with a Prolene mesh reversed restriction and restored synchrony during quiet breathing. However after surgery during exercise there was a significant reduction in the motion of upper rib cage compartment compared to preoperative values due defective expiratory mechanics on the operated side following reconstruction. This is further supported by a persistently reduced FEV_1_ after surgery.

## Conclusion

This physiological study demonstrates the negative effect of chest wall tumours on global chest wall mechanics during quiet breathing and exercise and describes how surgery in part reverses this abnormality. Further studies are required to look at the effects of rigid and physiological type repairs and to correlate findings with patients’ function and disability.

## Consent

Written informed consent was obtained from the patient for publication of this Case report and any accompanying images. A copy of the written consent is available for review by the Editor-in-Chief of this journal.

## Abbreviations

AB: The abdomen
EEV: End expiratory volume
FEV_1_: The forced expiratory volume in one second
Neg: Negative
OEP: Optoelectronic plethysmography
RCA: Lower rib cage
RCP: The upper rib cage (RCP)
